# *PTEN* Alterations and Their Role in Cancer Management: Are We Making Headway on Precision Medicine?

**DOI:** 10.3390/genes11070719

**Published:** 2020-06-28

**Authors:** Nicola Fusco, Elham Sajjadi, Konstantinos Venetis, Gabriella Gaudioso, Gianluca Lopez, Chiara Corti, Elena Guerini Rocco, Carmen Criscitiello, Umberto Malapelle, Marco Invernizzi

**Affiliations:** 1 Department of Oncology and Hemato-Oncology, University of Milan, 20122 Milan, Italy; konstantinos.venetis@unimi.it (K.V.); elena.guerinirocco@ieo.it (E.G.R.); 2 Division of Pathology and Laboratory Medicine, IEO, European Institute of Oncology IRCCS, 20141 Milan, Italy; elham.sajjadi@ieo.it; 3 Doctoral Program in Translational Medicine, University of Milan, 20133 Milan, Italy; 4 Division of Pathology, Fondazione IRCCS Ca’ Granda Ospedale Maggiore Policlinico, 20131 Milan, Italy; gabriella.gaudioso@policlinico.mi.it (G.G.); gianluca.lopez@unimi.it (G.L.); chiara.corti1@unimi.it (C.C.); 5 New Drugs and Early Drug Development for Innovative Therapies Division, IEO, European Institute of Oncology IRCCS, 20141 Milan, Italy; carmen.criscitiello@ieo.it; 6 Department of Public Health, University Federico II, 80138 Naples, Italy; umberto.malapelle@unima.it; 7 Department of Health Sciences, University of Eastern Piedmont, 28100 Novara, Italy; marco.invernizzi@med.uniupo.it

**Keywords:** *PTEN*, tumor suppressor, *PI3K/Akt*, cancer, solid tumors, tumor immune microenvironment, biomarker, precision medicine

## Abstract

Alterations in the tumor suppressor phosphatase and tensin homolog (*PTEN*) occur in a substantial proportion of solid tumors. These events drive tumorigenesis and tumor progression. Given its central role as a downregulator of the phosphoinositide 3-kinase (PI3K)/Akt/mammalian target of rapamycin (mTOR) pathway, *PTEN* is deeply involved in cell growth, proliferation, and survival. This gene is also implicated in the modulation of the DNA damage response and in tumor immune microenvironment modeling. Despite the actionability of *PTEN* alterations, their role as biomarkers remains controversial in clinical practice. To date, there is still a substantial lack of validated guidelines and/or recommendations for *PTEN* testing. Here, we provide an update on the current state of knowledge on biologic and genetic alterations of *PTEN* across the most frequent solid tumors, as well as on their actual and/or possible clinical applications. We focus on possible tailored schemes for cancer patients’ clinical management, including risk assessment, diagnosis, prognostication, and treatment.

## 1. Introduction

Phosphatase and tensin homolog (*PTEN*) is a key tumor suppressor that inhibits cell growth and enhances cellular sensitivity to apoptosis [1]. Since its first discovery in 1997, the role of *PTEN* as a biomarker in cancer has become more and more significant [2,3]. To date, alterations in this gene and/or protein expression are viewed as actionable molecular hallmarks, meaning that their presence has implications on clinical decision-making [4]. Loss or altered *PTEN* function has been identified in a wide spectrum of neoplasms, being considered a founder genetic event for tumorigenesis and tumor progression [5]. *PTEN* activity can be either dependent on or independent of the down/de-regulation of the phosphatidylinositol-3-kinase (*PI3K*)/protein kinase B (*Akt*) pathway [6]. Accumulating data suggest that *PTEN* is also implicated in the overall DNA damage response and in modeling the adaptive arm of the anti-tumor immune response [7,8,9]. The feasibility of identifying *PTEN* alterations in clinical practice is a matter of controversy, given the lack of companion and/or complementary diagnostic tests for their investigation. In addition, due to the three-dimensional level of alterations (i.e., DNA, mRNA, and protein expression) involving *PTEN*, the development and validation of a single testing approach covering all the clinically significant variations are intricate. Despite the fascinating insights provided by several studies over the past two decades, the multifaceted biology of *PTEN* remains not entirely understood. In this review article, we seek to outline the biological and genetic changes of *PTEN* in solid tumors, focusing on possible strategies for achieving tailored schemes for patients’ risk assessment, diagnosis, prognostication, and treatment.

## 2. Structure and Cellular Roles of *PTEN* Tumor Suppressor

The *PTEN* gene (10q23.31) comprises 9 exons and a variable additional exon (i.e., exon 5b) that is omitted in the major transcript [1]. A highly conserved sequence upstream of the promoter contains a canonic E-box sequence, which is involved in *PTEN* transcriptional activation [10]. *PTEN* is a dual-specificity protein phosphatase that is composed of 403 amino acids across five functional domains and has major enzymatic activity on phosphatidylinositol (3,4,5)-trisphosphate (PIP3) [1]. 

**Figure 1:**
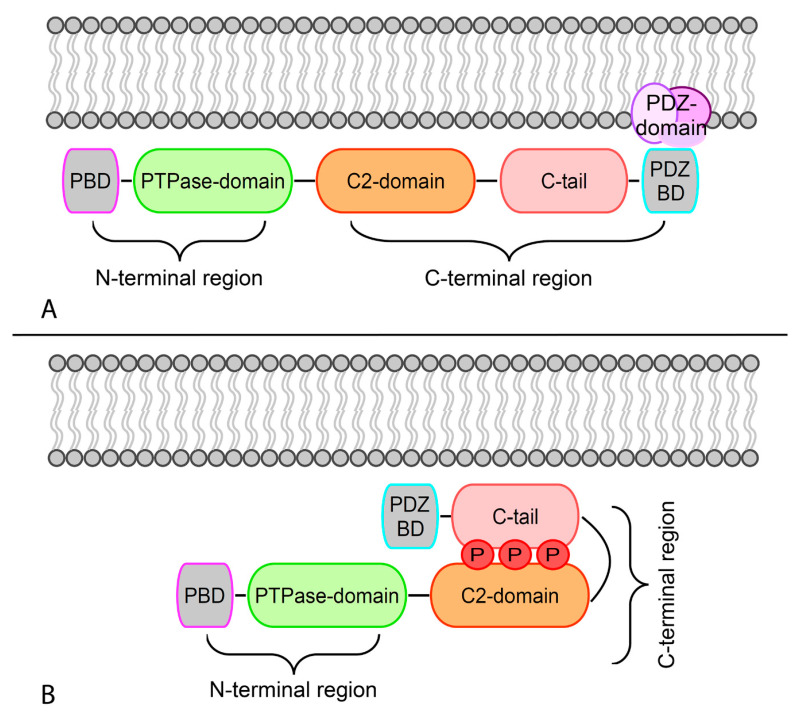
Schematic representation of the open and closed conformations of phosphatase and tensin homolog (PTEN) based on its phosphorylation status. (**A**) Dephosphorylation leads to the open conformation that allows PTEN to associate with the membrane. The association of PTEN with the negatively charged membrane occurs through electrostatic interactions. (**B**) Binding of the C2 domain to phosphatidylserine leads to a conformational change and activation of the phosphatase C-tail domain.

The first 14 residues at the N-terminal region constitute the phosphatidylinositol 4,5-bisphosphate (PIP2) binding domain (PBD) [11]. Several oncogenic mutations may target this region, resulting in a lowered affinity of *PTEN* for the cell membrane [1]. The protein tyrosine phosphatase (PTPase) domain represents the active site of *PTEN*. This wide and positively charged pocket region is able to accommodate the large, negatively charged PIP3 substrate. Located at the C-terminal region, the *PTEN* type II calcium-independent (C2) domain interacts nonspecifically with the plasma membrane. The complexity of the interaction between the PTPase and C2 domains, and the critical role in *PTEN* regulation, have led to their depiction as a “super-domain” [12]. Several driver mutations result in the breakdown of the PTPase-C2 boundary, with subsequent *PTEN* inactivation (Figure 1A). The *PTEN* C-terminal extremity is composed of a carboxyl-terminal tail (C-tail) and a PDZ-binding domain (PDZ-BD) that acts as a protein-protein interaction motif [5]. Premature stop codons that remove the C-tail are driver genetic events for tumorigenesis and tumor progression. The spectrum of possible post-translational modifications of the *PTEN* C-terminal region is exceedingly heterogeneous [1]. Phosphorylation in C2 and C-tail domains of the C-terminal region promotes their interaction, resulting in the “closed” conformation of the phosphorylated form of *PTEN* [1,13]. As a consequence of this modification, the interactions between PDZ-BD and PDZ domain-containing proteins in the plasma membrane are inhibited [14]. Auto-dephosphorylation reverses this conformational change to “open” status, allowing *PTEN* to bind to the membrane and PDZ domain-containing proteins (Figure 1). As a general rule, we can say that phosphorylation phenomena reduce *PTEN* activity by increasing its chemical stability. Other post-transcriptional modifications include ubiquitylation, oxidation, acetylation, and small ubiquitin-like modifier (SUMO)ylation.

### 2.1. PTEN as a Downregulator of the PI3K/Akt/mTOR Pathway

**Figure 2:**
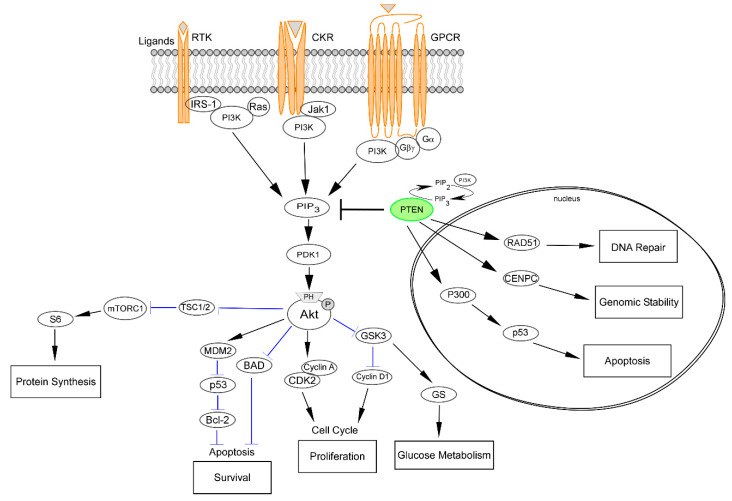
PI3K/Akt/mTOR signaling and its interaction with PTEN. The PI3K pathway regulates diverse cellular processes, including protein synthesis, cell survival, proliferation, glucose metabolism, apoptosis, DNA repair, and genome stability. Akt-mediated phosphorylation inhibits the activity of the TSC1-TSC2 complex, also known as hamartin-tuberin. This is a critical step for the negative regulation of mTORC1, whose activity controls anabolic processes. Another important downregulation of Akt phosphorylation is towards BAD, while MDM2 activity is enhanced, promoting the degradation of the tumor-suppressor p53, which also plays a part in the P300-mediated cell apoptosis. Cell cycle regulation occurs by means of cyclins A and D stimulation and GSK3 inhibition. The latter event is also responsible for increased glucose metabolism. PTEN is intimately involved in the regulation of these mechanisms through its substrate PIP3. Of note, the activity of PTEN in the cell nucleus that leads to cell survival control is related to the upregulation of key mediators, such as RAD51, CDNPC, and P300. RTK, receptor tyrosine kinase; CKR, chemokine receptor; GPCR, G protein-coupled receptor; IRS-1, insulin receptor substrate 1; PI3K, phosphatidylinositol 3-kinase; JAK1, Janus kinase 1; PIP3, phosphatidylinositol (3,4,5)-trisphosphate; PDK1, pyruvate dehydrogenase lipoamide kinase isozyme 1; TSC, tuberous sclerosis complex; mTORC1, mammalian target of rapamycin complex 1; MDM2, mouse double minute 2 homolog; BAD, BCL2 associated agonist of cell death; GSK3, glycogen synthase kinase-3; CDK2, cyclin-dependent kinase 2; CDNPC, centromere protein C.

The phosphoinositide 3-kinase (*PI3K*)/*Akt*/mammalian target of rapamycin (*mTOR*) is the most commonly upregulated pathway in human cancers [15]. It is profoundly involved in many aspects of cell growth, proliferation, survival, metabolism, and immune response regulation (Figure 2) [16]. Abnormal activating events targeting PI3K/Akt result in a deep disturbance of these processes, which ultimately leads to tumorigenesis, metastasis, tumor progression, and therapy resistance [17,18,19]. PI3Ks are grouped into three classes based on their substrate specificity and structure [20]. In particular, class I PI3Ks are signal transducers of tyrosine kinases, G protein-coupled receptors (GPCRs), and small GTPases, while class II and class III PI3Ks influence signaling indirectly by mainly regulating membrane trafficking [21]. Activation of G protein-coupled receptors (GPCRs), chemokine receptors (CKRs), and receptor tyrosine kinases (RTKs) on the plasma membrane by specific ligands results in autophosphorylation on tyrosine residues [16]. The three classes of PI3K are then recruited and activated, resulting in the production of the second messenger PIP3 from the substrate PIP2 [22]. This process leads to the recruitment of signaling proteins, including protein serine/threonine kinase-3′phosphoinositide-dependent kinase 1 (*PDK1*) and *Akt* [16,22]. This mechanism is the “core” of cell survival and cell cycle progression control. Following phosphorylation, the serine-threonine kinases are fully activated, either by PDK1, the mTOR complexes, or other kinases [23]. *Akt* is directly inactivated by the dephosphorylation from phosphatase domain and leucine-rich repeat protein phosphatase (*PHLPP*) [23]. On the contrary, the negative regulation of mTORC1 and PIP3 activity by *PTEN*, tuberous sclerosis 1 (TSC1), tuberous sclerosis 2 (TSC2), and liver kinase B1 (LKB1) lead to the indirect inactivation of Akt [24]. An important part of the *PTEN* tumor suppressor role is represented by the negative regulation of the *PI3K/Akt/mTOR* pathway (Figure 2). Loss of *PTEN* activity leads to the stable activation of the *PI3K/Akt* signaling, with subsequent abnormal cell growth, survival, and proliferation [25]. It is important to note, however, that an important part of the *PTEN* tumor-suppressor role is outside the *PI3K/Akt* axis [26].

### 2.2. Nuclear PTEN and Modulation of the DNA Damage Response

Once considered a strictly cytoplasmic protein, PTEN is now known to be present and active in the nucleus, mitochondria, endoplasmic reticulum, and extracellular space [27,28]. In particular, PTEN activity in the nucleus is critical for tumor suppression, independently of PTEN phosphatase activity [28,29,30]. However, there is an understanding gap between tumorigenesis and the dysregulation of PTEN nuclear functions. PTEN is localized within the centromere where it physically interacts with proteins that are essential for centromere formation and stabilization, such as centromere-specific binding protein C (CENP-C) [31]. The stability of the centromere is a result of RAD51 expression by PTEN modulation (Figure 2). Given that RAD51 is a key component of the double-strand breaks (DSB) homologous recombination (HR) DNA repair systems, PTEN is currently viewed as a DNA-damage response regulator [32,33]. Importantly, PTEN loss may lead to DSB also through increased Akt-mediated cytoplasmic sequestration of the checkpoint kinase 1 (CHK1), resulting in altered G2/S arrest in response to DNA damage [34]. In addition, PTEN plays a key role in regulating and maintaining the integrity of several checkpoints during the G1-S and G2-M cell cycle transitions [35,36]. Specifically, the intra-S checkpoint is an important DNA damage stage which stops cell cycle progression to allow repair of DNA damage [37]. Hence, radiation-induced DNA damage in the presence of an impaired PTEN activity has been associated with an accelerated transition from G2/M to G1 while PTEN phosphorylation leads to accelerated G2-M transition [36]. An additional nuclear function of PTEN is related to its interaction with p53 which subsequently leads to G1 arrest [38]. Another mediator of PTEN-related cell cycle control is represented by histone acetyltransferase (HAT) [39]. The interaction between PTEN and HAT is particularly noticeable in the control of chromatin dynamics and global gene expression [40]. Through its C-terminal domain, PTEN is able to bind histone H1 and to maintain a normal chromatin condensation [29,39]. The multiple functions of PTEN, the contribution across different cellular processes, and the interactions with numerous cellular components highlight the essential role that leads scientists to nominate it as a novel guardian of the genome [41].

**Figure 3:**
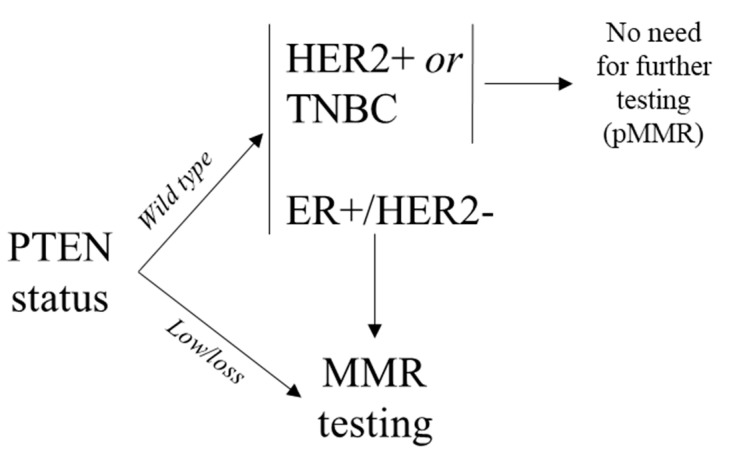
Diagnostic algorithm for the evaluation of mismatch repair status in breast cancer using PTEN immunohistochemistry as a complementary test. In this proposed workflow, PTEN can be profiled before the execution of other tests in order to pre-screen patients for MMR proficiency. Tumors showing HER2 overexpression and/or amplification and those with a triple-negative phenotype should be considered mismatch repair proficient if PTEN expression is retained, with a positive predictive value of 95% and 100%, respectively. TNBC, triple-negative breast cancer; ER, estrogen receptor; MMR, mismatch repair; pMMR, mismatch repair proficient.

Another important observation that corroborates the role of PTEN in regulating the DNA damage response is represented by its expression in endometrial tumors. Of note, loss of PTEN expression and microsatellite instability (MSI) are two of the more common molecular alterations in endometrial carcinoma [42]. Indeed, in the MSI hypermutated molecular subgroup, PTEN shows high mutation rates and decreased protein expression [43]. These alterations co-occur with those in phosphatidylinositol 4,5-bisphosphate 3-kinase catalytic subunit, alpha isoform (PIK3CA), both in the mismatch repair (MMR) deficient/MSI and the copy number-low subgroups. It has been hypothesized that the 5’-polyadenosine tracts in PTEN might be a target for mutations in MMR-deficient tumors. Hence, several studies have reported that many PTEN mutations associated with MSI-high (MSI-H) status occur with higher frequency in the poly(A_6_) regions compared to PTEN mutations found in microsatellite stable (MSS) tumors, particularly in endometrial cancer and colorectal cancer [44,45]. In an elegant study, Djordjevic and collaborators observed that in non-endometrioid endometrial carcinomas, a PTEN-retained and/or wild-type status significantly co-occur with a retained positive expression of the MMR proteins [46]. Similarly, significant correlations between MMR proficiency, PTEN wild-type expression, and a better outcome have been recently reported in breast cancers [47]. These observations can be of clinical value not only for Lynch syndrome screening (e.g., endometrial cancer, colorectal cancer) but also for prognostication and immunotherapy prediction (e.g., breast cancer). Indeed, the Food and Drug Administration (FDA) has approved in the recent past an immune checkpoint inhibitor compound (i.e., pembrolizumab) in MMR deficient solid tumors, irrespective of the tumor origin [48]. Regrettably, information on the specific biology of MMR deficiency in breast cancer is extremely limited in the literature. Genomic scars in the MMR system are relatively rare in breast cancer, being reported in ~2% of cases [49]. However, this subject is controversial in literature given the lack of companion diagnostics and/or tumor-specific guidelines for MMR analysis [47,49,50,51,52,53,54]. Unlike in endometrial and colorectal cancers, MSI is restricted to a minority of breast cancers showing MMR protein loss [54]. Of note, MMR but not PTEN proteins expression by immunohistochemistry (IHC) show a remarkable degree of intra-tumor heterogeneity, resulting in a possible diagnostic algorithm to overcome this issue, as shown in Figure 3. 

### 2.3. The Interplay between PTEN and the Tumor Immune Microenvironment

**Figure 4:**
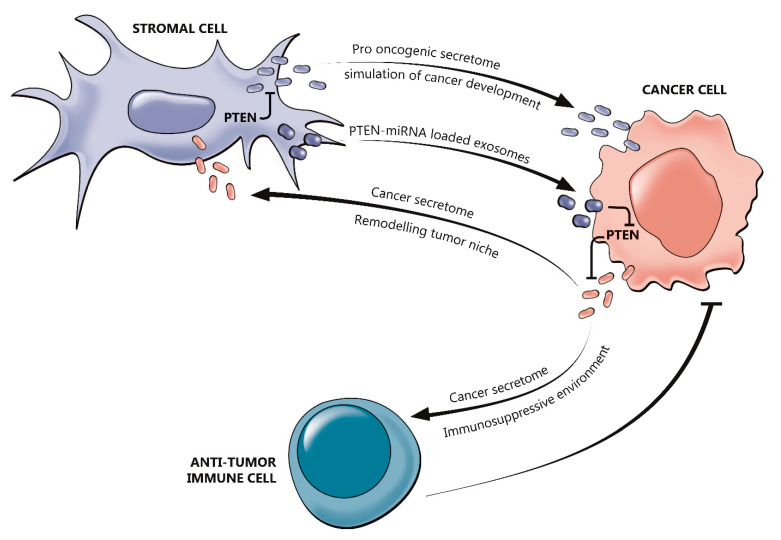
Role of PTEN in the regulation of the tumor immune microenvironment. PTEN in tumor cells may regulate the cancer cell secretome to prevent the secretion of immunosuppressive chemokines and, consequently, favoring the establishment of an immune-permissive tumor microenvironment, which would improve antitumor immune responses. PTEN may also prevent the formation of reactive stroma with pro-tumorigenic activity, whereas in stromal cells may suppress tumorigenesis through inhibition of a pro-oncogenic secretome that reprogrammes cancer cells. Cells in the tumor microenvironment may produce exosomes that contain PTEN-targeting microRNA (miRNA) to downregulate the expression of PTEN in cancer cells, thereby counteracting the tumor-suppressive effects of PTEN.

The overall anti-tumor immunosuppressive activity relies in part on *PTEN* regulation [9]. Several studies have demonstrated that *PTEN* acts as downstream of T- and B-cell receptors, as well as in negatively regulating the expression of the immunosuppressive cytokine (e.g., interleukin (IL)-10) and vascular endothelial growth factor (VEGF) [55]. Additionally, *PTEN* can affect cytokine-induced responses of the IL-2 receptor through the Akt signaling [56]. This is a critical step in the modulation of regulatory T cells (Tregs) activity [56]. In B-cells, this negative regulation influences the proliferation, activation, and survival [55]. Given these considerations, it is not surprising that loss of *PTEN* activity is associated with cellular and humoral immune dysfunction and autoimmunity [9,34]. The cancer secretome, which is composed of macromolecules secreted by tumor cells, may stimulate the expression of immunosuppressive cytokines (e.g., IL-6 and IL-10) [57]. It has been observed that the lack of *PTEN* leads to the expression of chemoattractant cytokines, such as chemokine (C-X-C motif) ligand 1 (*CXCL1)*, granulocyte colony-stimulating factor (*G-CSF)*, and *IL-23*. [58]. Along with their activity in suppressing anti-tumor immunity, cytokines can affect the activation of both infiltrating inflammatory cells and stromal cells [57]. In this scenario, several growth factors are also produced to enhance PI3K pathway harvest in PTEN deficient cells, thus sustaining tumor growth [58]. In summary, different tumor microenvironments could be a result of different patterns of *PTEN* loss in different molecular scenarios. This was noted by Bezzi et al. as they found out that combined deletion of *PTEN* and Zinc Finger BTB Domain Containing 7A (Zbtb7a) gene in prostate cancer results in tumor progression, while combined *PTEN* and p53 loss are related to immunosuppression [59]. Genetic inactivation of PTEN facilitates the initiation, progression, and malignant transformation of epithelial tumors. Tumor growth and invasiveness are inhibited by normal fibroblasts, in contrast, reprogrammed fibroblasts which co-evolve with epithelial tumor cells are thought to induce tumor initiation and progression [60,61,62]. In *PTEN* low breast cancers, both mRNA and microRNA (miR) expression can be reprogramed in normal fibroblasts, leading to a tumor-associated fibroblast phenotype. Moreover, *PTEN* loss may lead to gene expression reprogramming in the mammary gland microenvironment by activated oncogenic secretome. miR-320 as a target of *PTEN* in stromal fibroblasts leads tumor microenvironment towards defeating aggressive phenotypes of breast cancer. Downregulation of this micro RNA in *PTEN*-deleted stromal fibroblasts in the oncogenic secretome stimulates tumor invasiveness and the creation of angiogenic networks [60]. The interactions between *PTEN* and tumor immune microenvironment are outlined in Figure 4.

## 3. *PTEN* Molecular Aberrations Clinical Roles 

### 3.1. Biomarker in Molecular Epidemiology

**Figure 5:**
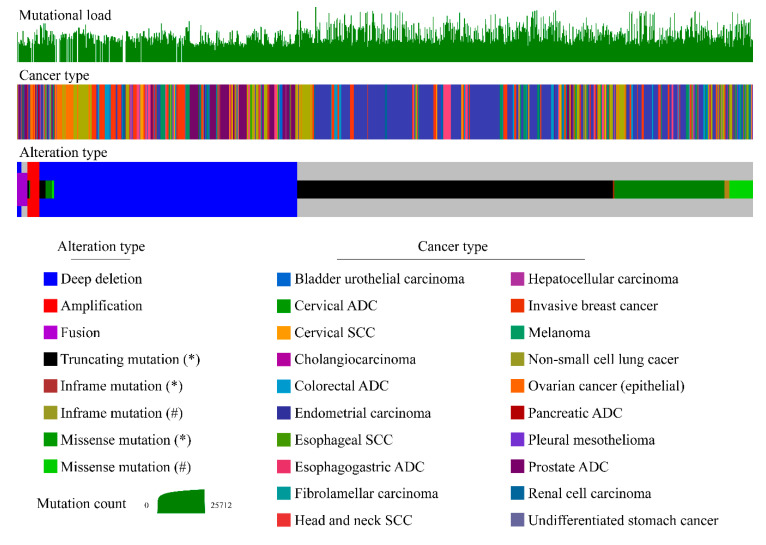
Oncoprint visualization of PTEN somatic molecular alterations overall mutational burden across PTEN-altered solid tumors. Types of alterations and tumors are color-coded on the basis of the legends on the bottom. Each column represents a sample and was sorted to appreciate the magnitude of alteration types. The bar plot on the top represents the mutation count, as reported on the green scale. The 19 main tumor types included in this analysis from cbioportal.org are melanoma, head and neck squamous cell carcinoma, non-small cell lung cancer (i.e., squamous cell and adenocarcinoma), mesothelioma, esophageal cancer, stomach cancer, colorectal cancer, cholangiocarcinoma, pancreatic cancer, liver cancer, renal cell carcinoma, bladder cancer, prostate adenocarcinoma, uterine cancer (i.e., endometrioid, serous, and carcinosarcoma), cervical cancer, ovarian cancer, and invasive breast cancer (samples *n* = 1068/7888; 13.5%). ADC, adenocarcinoma; SCC, squamous cell carcinoma.

PTEN altered function occurs in approximately 13.5% of human cancers through inframe, missense, and truncating mutations, gene fusions, amplifications, deletions, epigenetic silencing, and transcriptional modifications (Figure 5) [1].

In particular, PTEN gene alterations are more frequent in endometrial cancer (35%), glial tumors (32%), prostate cancer (17%), melanoma (13%), non-small small-cell lung cancer (NSCLC) (12%), and breast cancer (9%) [63,64,65,66,67,68]. Of note, PTEN protein loss is a more frequent event in cancer, compared to PTEN genetic alterations, particularly in lung cancer and breast cancer [42,47,69]. Hence, only 9% of lung squamous cell carcinoma harbor PTEN somatic mutations, while up to 44% showed decreased protein levels [69]. Similarly, PTEN protein loss or low expression occurs in 46% of invasive breast cancers, with particularly higher frequency in ductal and estrogen receptor (ER)^POS^/HER2^NEG^ breast cancers [47]. On the other hand, mutation, loss of heterozygosity (LOH), and methylation account for 5%, 40%, and 50% of cases respectively [29,70]. Functional PTEN loss in endometrial carcinoma can be also mediated by a number of other mechanisms, including PTEN gene regulation by miRNAs, and alterations of protein stability [46,64]. Loss of PTEN expression recurs with high frequency in gliomas and glioblastomas. Furthermore, inactivation of PTEN both by mutations and by epigenetic silencing plays an important role in the development of cutaneous melanoma [29,71]. PTEN loss or inactivating mutations are found in a variable proportion (5–30%) of sporadic colorectal cancers [72,73,74]. Up to 18% of colorectal cancer harbors PTEN point mutations and up to 19% LOH, depending on tumor type, and concomitant promoter hypermethylation [29,75]. 

**Figure 6:**
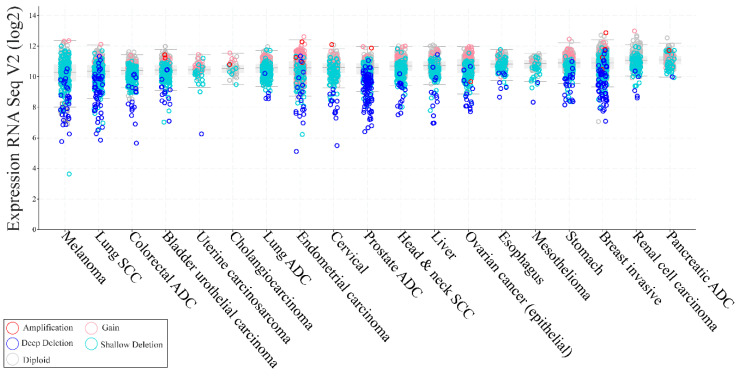
RNA expression levels and copy-number alterations of PTEN across PTEN-altered solid tumors. Each column represents a tumor type from cbioportal.org datasets. Types of alterations are color-coded on the basis of the legend on the bottom. ADC, adenocarcinoma; SCC, squamous cell carcinoma.

In sporadic tumors, PTEN LOH occurs at a higher frequency than biallelic inactivation [76]. However, it is still not clear whether haploinsufficiency provides a selective growth advantage in tumors lacking a second hit in the remaining PTEN allele [77]. The observation that PTEN loss alone is sufficient to cause tumorigenesis in some tissue types but not in others, making the role of PTEN more ambiguous [29,78]. In addition to genetic loss or mutations, alterations in RNA expression are frequent in solid tumors, albeit heterogeneous (Figure 6). 

Overexpression of PTEN has been a matter of interest both in terms of biologic and clinical significance. Studies conducted in NSCLC cell lines have reported that overexpression of PTEN was associated with inhibition of tumor growth [79] and an increase in levels of cleaved caspase-3 and cell arrest in G0/G1 phase, suggesting its role as a potential target [80]. In these patients, improvement in the efficacy of pemetrexed, a first-line chemotherapy drug, have been observed [81]. Several studies using different types of cancer cell lines have shown that PTEN overexpression can increase apoptosis in glioblastoma [82], inhibit proliferation and promote apoptosis of hepatocytes [83], improve cisplatin-resistance of ovarian cancer by upregulating a downstream molecule named as keratin, type I cytoskeletal 10 (KRT10) [84], and having a cooperative role with lithium for reducing colorectal cancer, suggesting their potential combination as a novel treatment [85]. In this respect, many mechanisms are capable of transcriptional and post-transcriptional regulation of PTEN expression, including epigenetic silencing, transcriptional repression, regulation by miRNAs, and disruption of competitive endogenous RNA (ceRNA) networks, which have all been shown to contribute to regulating PTEN levels [5]. Moreover, PTEN is subject to a wide range of post-translational modifications that ultimately governs its protein levels, activity, and function, including overexpression of PTEN-interacting proteins, dimerization, and secretion [5]. Because insulin-mediated metabolic responses are primarily achieved through PI3K, PTEN haploinsufficiency has been demonstrated to be a monogenic cause of profound constitutive insulin sensitization that is apparently obesogenic [86]. Patients who are heterozygous carriers of PTEN mutations, seem to have an increased risk of obesity and cancer, but a decreased risk of diabetes due to enhanced insulin sensitivity [29,86].

### 3.2. Phenotypic Variability in PTEN Germline Mutation Carriers and Genotype-Phenotype Relationship

PTEN germline mutations have been extensively studied in patients affected by Cowden disease, an autosomal dominant condition characterized by hamartomas and increased lifetime risk of several types of cancers (e.g., breast, thyroid, endometrium) [87]. Since then, several other PTEN-related hamartoma tumor syndromes (PHTS) have been identified [88]. These patients show multiple hamartomas in a variety of anatomic sites and have characteristic cutaneous manifestations, such as trichilemmomas, oral fibromas, and punctate palmoplantar keratoses [89]. According to Tan et al. and Bubien et al., these patients present an increased estimated lifetime risk of breast cancer of around 80%, while the lifetime risk for thyroid cancer is approximately 30% [90,91]. The lifetime risks for endometrial cancer, colorectal cancer, and renal cell carcinoma were 28%, 9%, and 34% respectively [91]. A more recent study showed that the frequency of PTEN germline mutations in breast cancer ranges between 0.05% and 0.2% [92]. Interestingly, breast cancers in patients carrying a PTEN germline mutation have been found to be mostly of ductal histotype, with the presence of apocrine differentiation [93]. Bannayan–Riley–Ruvalcaba syndrome is another rare PHTS which is clinically characterized by multiple subcutaneous lipomas, macrocephaly, and penile lentigines [87]. In this condition, an increased risk for malignant tumors has not been demonstrated yet. Similar mutation patterns in PTEN can be observed in both Cowden and Bannayan–Riley–Ruvalcaba syndromes, suggesting that these two autosomal dominant disorders are related to allelic disorders [89]. Lhermitte–Duclos disease is characterized by hamartomatous outgrowths of the cerebellum (cerebellar dysplastic gangliocytoma) [94,95]. This PHTS may also arise in conjunction with Cowden syndrome [91]. Two other conditions have been associated with PTEN mutations and, although they lack hamartomas, some argue that they should be incorporated into the definition of PHTS [29]. The first condition is a Proteus-like syndrome with a distinct type of epidermal nevus (also referred to as segmental overgrowth, lipomatosis, arteriovenous malformation, and epidermal nevus (SOLAMEN) syndrome or type 2 segmental Cowden syndrome) that likely relates to the early LOH at the PTEN allele in the affected tissues in patients with underlying germline PTEN mutation [96,97,98]. The second condition is an autism spectrum disorder with microcephaly [99]. Published data on clinical manifestations come mainly from small case series or compilations of published cases, most of which predated the development of consensus criteria for diagnosis and genetic testing for Cowden syndrome [100]. As a result, the true frequencies of the clinical features of Cowden syndrome are not known and, as a consequence, it is difficult to perfectly describe genotype-phenotype correlations [89]. Some phenotypic analyses of small clinical cohorts of PTEN mutation carriers, combined with laboratory studies of the consequences of these mutations imply that stable catalytically inactive PTEN mutants may lead to the most severe phenotypes and to a higher number of lesions and, conversely, that mutants retaining partial function or with truncating mutations associate more frequently with a milder phenotype, with autism spectrum disorder often being diagnosed [87,101]. Albeit debated, genetic screening is currently available for other genes that have been associated with a Cowden-like presentation, including RAC-alpha serine/threonine-protein kinase (AKT1), killin (KLLN), PIK3CA, and succinate dehydrogenase complex (SDH) (subunits B and D) [87]. 

### 3.3. Inactivating Mutations of PTEN in Non-Familial Solid Tumors

**Figure 7:**
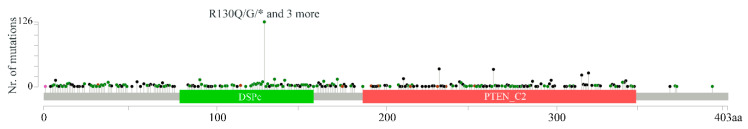
Type of mutations, frequency, and affected PTEN domains across selected solid tumors. The 19 tumor types included in this analysis from cbioportal.org are melanoma, head and neck squamous cell carcinoma, non-small cell lung cancer (i.e., squamous cell and adenocarcinoma), mesothelioma, esophageal cancer, stomach cancer, colorectal cancer, cholangiocarcinoma, pancreatic cancer, liver cancer, bladder cancer, prostate adenocarcinoma, uterine cancer (i.e., endometrioid, serous, and carcinosarcoma), ovarian cancer, and invasive breast cancer. Green, missense; black, truncating; brown, inframe; purple, other types of genetic alteration.

According to the Catalogue of Somatic Mutations in Cancer (COSMIC), 1.993 unique somatic PTEN mutations have been found in human cancers [5]. Both germline and somatic PTEN mutations have been identified in the promoter and all nine exons of PTEN (Figure 7), including missense, nonsense, splice site variants, intragenic deletions and insertions and large deletions [5]. 

**Figure 8:**
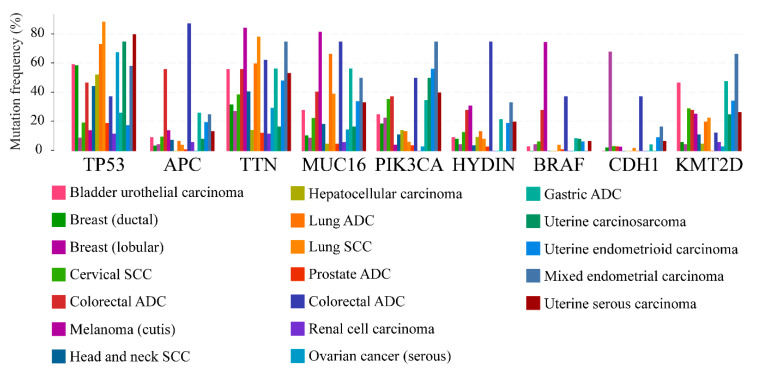
Frequency of mutations targeting the highly recurrent mutated genes in PTEN-defective solid tumors. Each tumor type is color-coded on the basis of the legend on the bottom. ADC, adenocarcinoma; SCC, squamous cell carcinoma.

These genetic aberrations lead to unstable truncated proteins that are almost undetectable and thus functionally comparable to the PTEN monoallelic loss [5]. The diversity of tumor-associated mutations occurring in all domains of PTEN strongly suggests that these different domains are physiologically relevant to PTEN-related tumorigenesis [76]. The majority of the missense mutations occur in the phosphatase domain and affect the catalytic activity of PTEN [102]. However, a broad spectrum of mutations in PTEN that are associated with primary tumors occurs outside the phosphatase domain, confirming additional activities of PTEN function in tumor suppression [103]. For example, nonsense and frameshift mutations yield truncated PTEN proteins lacking the C-terminal tail and the PDZ-interaction motif [76]. Among the hotspot mutations, the most frequent are those at position 130, in the phosphatase domain of the protein. In the MSK-IMPACT Clinical Sequencing Cohort, they occurred 71 times, including 3 duplicate mutations in patients with multiple samples. 33 mutations appeared to cause R130Q substitutions (c.389G>A), 15 alterations determined R130G substitutions (c.388C>G), 19 mutations identified (c.388C>T) originated R130* amino acids loss due to the introduction of a premature stop codon, 2 changes caused R130P substitutions and 2 alterations were R130Qfs*4 (c.389delG), thus resulting in a frameshifting change with Arginine-130 as the first affected amino acid, replacing it for a Glutamine and creating a new reading frame ending at a stop at position 4 (counting starts with the Glutamine as amino acid 1). The C2-domain is involved especially in truncating mutations and the most frequent ones are located at positions 233, 267, and 319–323. Of note, codons 321–323 in exon 8 are characterized by a poly(A_6_) stretch. A pioneering study found out that PTEN mutations, besides being frequent in endometrial carcinomas (21/38, 55%), are associated with MSI. Hence, 14/18 (78%) of MSI-H tumor were *PTEN* mutated versus 7/20 (35%) of MSS cases. Interestingly, 6 tumors, 4 of which were MSI-H, had either an insertion or a deletion within a poly(A_6_) stretch in codons 321–323 [104]. PTEN deficiency could be a result of germline or somatic mutations, epigenetic silencing, abnormal transcriptional regulation, post-translational modifications, and protein-protein interactions [105,106]. The most frequently mutated genes in PTEN-defective solid tumors are well-known cancer genes, such as TP53, adenomatous polyposis coli (APC), titin (TTN), mucin 16 (MUC16), PIK3CA, axonemal central pair apparatus (HYDIN), BRAF, cadherin-1 (CDH1), and histone-lysine N-methyltransferase 2D (KMT2D), irrespective of the tumor type (Figure 8).

## 4. Clinical Actionability of PTEN Alterations

Prior to the massively parallel sequencing era, PTEN has been one of the most deeply sequenced tumor genes, with a vast amount of data to identify connections between carcinogens and tumorigenesis generated. However, since its first discovery by two independent research groups more than 20 years ago, PTEN has been extensively studied but not exhaustively. In the precision medicine era, huge efforts have been made to characterize tumors and tailor treatments according to genetic, epigenetic, or proteomic alterations. The integration between translational and clinical research is crucial in understanding tumor biology and, accordingly, identifying potential biomarkers of drug response or resistance. Several studies investigating mechanisms of resistance in breast cancer patients treated with PI3K inhibitors or CDK4/6 inhibitors have found interesting results regarding the implication of PTEN in the development of resistance. In one of these studies, patients who received PI3Kα inhibitors due to PIK3CA mutations and developed resistance after an initial response [107]. Convergent loss of PTEN expression was observed in the post-treatment metastatic samples when compared to the pre-treatment tumor, suggesting the potential role of PTEN in the development of resistance. The authors conclude that loss of PTEN expression might be due to selective therapeutic pressure [107]. A recent study conducted on ER^POS^ advanced breast cancer patients treated with a combination of the CDK4/6 inhibitor ribociclib and letrozole showed that loss of PTEN expression due to AKT activation could lead to the development of resistance to CDK4/6 inhibition [108]. Along with the observation that PTEN loss promotes resistance to PI3Kα inhibitors, the authors highlight the possibility that one genetic event might prove sufficient for the same patient to develop clinical cross-resistance to multiple targeted therapies [108]. The potential use of PTEN loss in predicting resistance to the PI3Kα inhibitor alpelisib has also been studied on liquid biopsies, with encouraging results [109]. 

PTEN is one of the most frequently altered genes in human cancer. The analysis of publicly available genomic datasets confirms that the overall mutational loads in cancers parallel those that can be found in PTEN itself. Thus, translational studies on PTEN alterations are still needed to broaden our understanding of the gene-environment interactions in cancer. In terms of clinical applications, PTEN alterations are extremely complex biomarkers. On the other hand, PTEN clinical studies have not led to a deep impact on cancer management and therapy yet. The complexity that underpins PTEN alterations and its interactions with other genes, pathways, biological systems and tumor microenvironment may have been responsible for the somehow nihilistic view of our ability to successfully generate clinically relevant biomarkers. Available evidence suggests an association between PTEN functional status and both clinical outcome and response to several therapies. However, its real prognostic and/or predictive role in cancer is still unknown. This can be, at least partially, due to the fact that PTEN assessment lacks consistency and reproducibility across different studies in terms of the type of assay, antibodies used for immunohistochemistry testing, scoring system, cutoffs for PTEN-loss/low definition, and source of tumor samples (primary versus metastatic). Moreover, the remarkably heterogeneous cohorts of patients in which the clinical utility of PTEN loss has been explored makes the interpretation of available results even more complex. Finally, it has been recently observed that PTEN might be implicated in the control of tumor microenvironment and immune system, thus fostering future potential development for an immunotherapy-based approach.

## 5. Concluding Remarks

Translating PTEN genomic scars into the clinics has begun but it is still not ready for prime time. When clinical studies will be designed considering PTEN as a biomarker, in particular as a predictive biomarker for oncologic patients’ therapy selection, the identification of the specific alteration assessed will be crucial. From this fascinating perspective, it will be really important considering that PTEN, like MET and other complex genes, can be targeted by a constellation of various alterations, at different biological levels (i.e., DNA, RNA, and protein). To fully exploit the role of the different types of gene alterations in terms of patient selection, it would be required to implement a new 3D biology approach, aim to simultaneously analyze the presence and the clinical–biological function of PTEN DNA, RNA and protein alterations. Large, multicentric clinical trials coupled with companion and/or complementary diagnostic tests, in which patients are thoroughly profiled for their PTEN status, would lead us a step closer to the full implementation of PTEN analysis in clinical practice. Albeit currently unrealized, the potential of PTEN as a biomarker in cancer remains promising and deserves further investigation. 

## 6. Patents

The MMR diagnostic algorithm and testing method reported in paragraph 4 is the subject of a patent application (#IT102018000010730) by three of the Authors (N.F., C.Co., and G.L.).
